# BMPR2 spruces up the endothelium in pulmonary hypertension

**DOI:** 10.1007/s13238-015-0208-7

**Published:** 2015-09-15

**Authors:** Jianhua Xiong

**Affiliations:** Center for Molecular Medicine, National Heart, Lung and Blood Institute, National Institutes of Health, Bethesda, MD 20892 USA

Pulmonary hypertension (PH) is a potentially lethal disorder because of a dearth of effective therapeutic options (Schermuly et al., 2011; Mehari et al., 2014). Pulmonary arterial hypertension (PAH) is a major type of PH that is defined by a mean pulmonary arterial pressure higher than 25 mm Hg at rest or 30 mm Hg during exercise (Kovacs et al., 2009). The majority of known genetic variations associated with PAH occur in bone morphogenetic protein receptor, type II (BMPR2), a type of transforming growth factor (TGF)-β family of receptors. BMPR2 mutations are responsible for the etiology of approximately 80% familial PAH and 30% idiopathic PAH (International P.P.H.C et al., 2000; Machado et al., 2006; Soubrier et al., 2013). Recent translational studies involving modulation of endothelial BMPR2 signaling have provided novel insights into treatment of PH, triggering a paradigm shift in our understanding of PH therapeutics (Fig. 1) (Long et al., 2015; Nickel et al., 2015; Prewitt et al., 2015).

**Figure 1:**
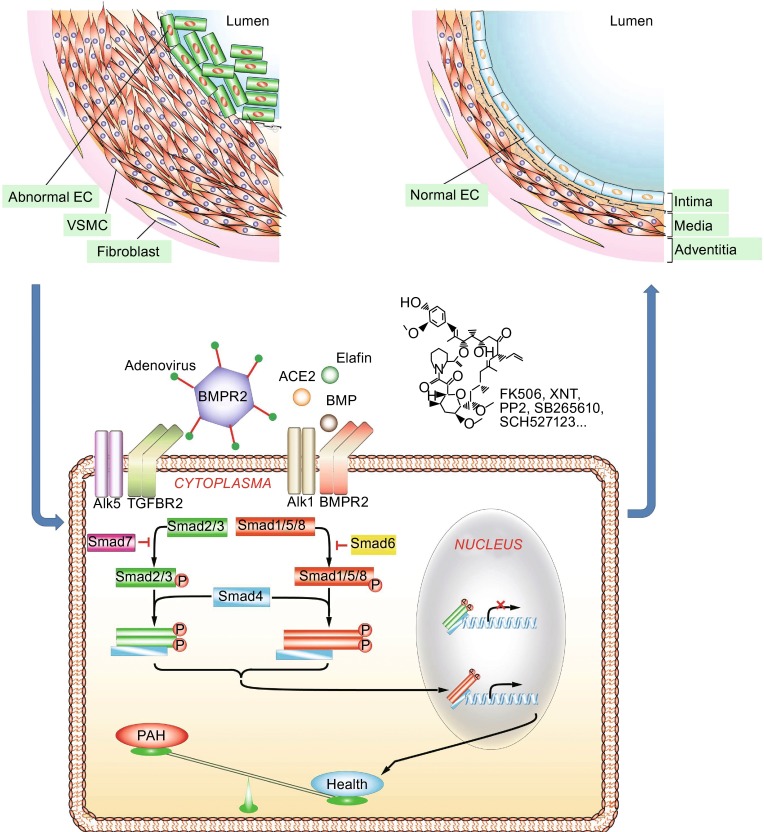
**Schematic illustration of potential endothelial BMPR2 signaling-related therapeutic approaches in pulmonary hypertension**. EC, endothelial cell; VSMC, vascular smooth muscle cell

At the 5th World Symposium on PH held in 2013, an updated clinical classification of PH was agreed upon (Simonneau et al., 2013). The current classification categorizes PH into five groups sharing similar pathophysiological characteristics and treatment approaches: Group 1, PAH; Group 1′, pulmonary veno-occlusive disease and/or pulmonary capillary hemangiomatosis; Group 1′′, persistent PH of the newborn; Group 2, PH due to left heart disease; Group 3, PH due to lung diseases and/or hypoxia; Group 4, chronic thromboembolic PH; Group 5, PH with unclear multifactorial mechanisms (Simonneau et al., 2013). Although PAH is a relatively rare disease, many genetic risk factors can substantially enhance its incidence and prevalence with an increased mortality (Peacock et al., 2007; Schermuly et al., 2011; Mehari et al., 2014). This is exemplified by the identification of over 300 mutations of BMPR2, which account for approximately 80% of patients with heritable PAH and 25% of patients with idiopathic PAH (Soubrier et al., 2013; West et al., 2014).

BMPR2 encodes a member of the TGF-β superfamily that operates in the TGF-β/bone morphogenetic protein (BMP) signal transduction pathways (International P.P.H.C et al., 2000; Soubrier et al., 2013). Intriguingly, pulmonary BMPR2 expression is over expressed in vascular endothelium, implying that BMPRS plays a key role in endothelial dysfunction underlying the development of PAH (Atkinson et al., 2002). As expected, heterozygous or homozygous BMPR2 ablation in mouse pulmonary endothelium leads to PAH (Hong et al., 2008). BMPR2 haploinsufficiency is involved in the pathobiology of PAH (Machado et al., 2001). Conditional endothelial-specific expression of BMPR2 mutations in mice induces a variety of PAH-related features including altered pulmonary microvascular endothelial cell (EC) apoptosis, proliferation, inflammation and thrombosis (Majka et al., 2011). Therefore, it is rational to consider activating and/or restoring a physiological balance of BMPR2 signaling for optimal treatment of PAH.

To assess the efficacy of BMP ligands in selectively targeting endothelial BMPR2 signaling, Long and colleagues generated a BMPR2-deficient mouse PAH model and examined two rat PAH models in response to either monocrotaline or vascular endothelial growth factor receptor blockade and hypoxia (Sugen-hypoxia) (Long et al., 2015). Initial results demonstrated that administration of BMP9 is capable of reversing PAH in these rodents. Consistent with this, enhancement of endothelial BMPR2 signaling by BMP9 is highly effective in preventing apoptosis and maintaining barrier integrity of pulmonary arterial endothelial cells (PAECs) from PAH patients bearing BMPR2 mutations. This lends further support to the idea that manipulation of BMPR2 signaling is a promising clinical strategy for the treatment of PAH (Long et al., 2015). Another BMP ligand, BMP2 has a comparable performance for stimulation of proliferation and induction of angiogenesis in PAECs. BMP2-mediated BMPR2 signaling requires both canonical and non-canonical Wnt pathways (de Jesus Perez et al., 2009). BMP7 and BMP9 also reduce apoptosis in human pulmonary microvascular ECs through up-regulation of alpha-B-crystallin, a process that is modulated by the BMPR2-ALK1 pathway (Ciumas et al., 2013). Moreover, using human pulmonary microvascular ECs and two conventional rat PAH models with either chronic hypoxia or monocrotaline treatment, Reynolds and co-workers showed that targeted adenoviral BMPR2 gene delivery displays success in attenuation of PAH properties (Reynolds et al., 2007, 2012).

In addition to direct targeting of BMPR2 pathway, specific manipulation of some BMPR2 signaling-related RNAs, metabolites, chemokines and enzymes still holds great promise in treatment of PAH. Interleukin-6-induced microRNA cluster 17/92 post-transcriptionally suppresses BMPR2 expression in human PAECs, indicating the potential use of microRNA inhibitors as therapeutic drugs (Brock et al., 2009). As BMPR2-deficient mice with chronic infusion of serotonin are susceptible to development of PH, inhibitors of serotonin ameliorate the pathology of PAH (Long et al., 2006). Downregulated BMPR2 expression stimulates translation of the chemokine granulocyte macrophage colony-stimulating factor (GM-CSF) and recruitment of macrophages in human PAECs, leading to inflammation-associated exacerbation of PAH. Nevertheless, GM-CSF-neutralizing antibody can subvert this deleterious process in PAH pathogenesis (Sawada et al., 2014). A lifetime risk of PAH development in a BMPR2 mutation-bearing individual is no more than 20% with a gender bias of female to male ratio of around 2.5:1. This incomplete penetrance of PAH suggests multi-faceted genetic, epigenetic and/or environmental factors may affect disease expression (Ma and Chung, 2014; Austin and Loyd, 2014). Particularly, variations in estrogen metabolism have been proposed to be responsible for the female predominance of PAH (Austin et al., 2009). Transcription factor estrogen receptor α-mediated BMPR2 suppression could be disrupted to elevate BMPR2 expression in female patients with PAH (Austin et al., 2012). BMPR2 mutation-related risk of PAH is correlated with increased estrogen metabolite 16α-hydroxyestrone and reduced metabolite 2-methoxyestrogen (Fessel et al., 2011). In pulmonary endothelium, intracellular BMPR2 cytoplasmic domain co-localizes and interacts with Tctex-1, a light chain of the motor complex dynein. Dysregulation of this interaction could interfere with a cascade of phosphorylation events, and thus act as a driver of PH (Machado et al., 2003). In addition, disrupted interaction of BMPR2 tail domain with cytoskeletal regulator LIMK1 may contribute to the etiology of PH via regulation of actin dynamics (Foletta et al., 2003). Cytoskeletal defects have also been described in BMPR2 mutant-expressing mouse pulmonary microvascular ECs (Johnson et al., 2012). Notably, Nickel and co-workers carried out an independent analysis of Sugen-hypoxia-induced severe rat PH model and PAECs from patients with PH. The results showed that the endogenous elastase antagonist elafin reverses the obliterative vascular remodeling *in vivo* and promotes the angiogenesis and survival of PAECs *in vitro* by amplifying BMPR2 signaling (Nickel et al., 2015). Moreover, exogenous recombinant human angiotensin-converting enzyme 2 (ACE2) can be used to correct BMPR2 signaling to normalize the pulmonary pressure in these PAH mice (Johnson et al., 2012).

The effect and efficiency of small chemicals have also been tested in reversal of PAH. Similarly to the effects of elafin, the synthetic activator of ACE2, XNT (1-[(2-dimethylamino) ethylamino]-4-(hydroxymethyl)-7-[(4-methylphenyl) sulfonyl oxy]-9H-xanthene-9-one) restricts pathogenic progression of monocrotaline-treated PAH rats (Ferreira et al., 2009). Recently, Prewitt et al. showed the SRC kinase inhibitor PP2 (3-(4-chlorophenyl)-1-(1,1-dimethylethyl)-1Hpyrazolo[3,4-d]pyrimidin-4-amine) can rescue the heterozygous null BMPR2 mutations-induced caveolar trafficking disorders with restoration of endothelial barrier function in pulmonary ECs (Prewitt et al., 2015). In addition, a group of chemical chaperones including thapsigargin, glycerol and sodium 4-phenylbutyrate, have been applied to facilitate the cell-surface trafficking of BMPR, suggesting therapeutic potential of these chemicals in endothelial BMPR2 rescue (Sobolewski et al., 2008). In an effort to understand why fewer than half of BMPR2 mutation carriers develop PH, Burton and colleagues revealed the inhibitory role of BMPR2 in inflammation which protects the PAEC barrier function in a CXCR2-dependent way during the course of PAH pathogenesis. Furthermore, the CXCR2 antagonist SB265610 and the CXCR1/2 antagonist SCH527123 are sufficient to dampen this phenotype with reduced leukocyte transmigration through endothelium and recovery from PAH (Burton et al., 2011a, b). BMPR2 mutations have been linked to enhanced cell apoptosis, inhibited cell proliferation and suppressed nitric oxide synthesis in human pulmonary microvascular ECs (Wang et al., 2014). Further investigation reveals that BMPR2 is required for the antiapoptotic drug effects of fluoxetine in monocrotaline-induced apoptosis of rat ECs (Wang et al., 2011). To systematically identify chemicals that can rescue the BMPR2 signaling axis, a transcriptional high-throughput luciferase reporter assay was performed using a Food and Drug Administration-approved pooled library of 3756 drugs and bioactive compounds. The preferred low-dose FK506 (tacrolimus) robustly reverses PAH in a conditioned endothelial BMPR2 knockout PAH mouse model and two rat PAH models exposed to either monocrotaline or Sugen-hypoxia. As anticipated, FK506 significantly improves the endothelial injury in PAEC derived from patients with idiopathic PAH (Spiekerkoetter et al., 2013).

Taken as a whole, the emerging roles of endothelial BMPR2 in treatment of PAH highlight the potential BMPR2 signaling-based therapeutic approaches. Although studies performed during the past 15 years have revealed a wealth of details about the molecular and cellular mechanisms underlying endothelial BMPR2 signaling, recent experimental advances are still unravelling an unexpected and encouraging diversity of this signaling. For instance, Diebold et al. demonstrated that BMPR2 is essential for maintenance of normal mitochondrial metabolism and DNA integrity and represses apoptosis of PAECs (Diebold et al., 2015). Using RNA sequencing analysis, Rhodes and colleagues found a novel pathway involving downregulation of endothelial COL4 and EFNA1 that underlies BMPR2-related endothelial dysfunction in PAECs (Rhodes et al., 2015). Metabolomic analysis reveals that a wide range of metabolic abnormality is associated with BMPR2 mutations in human pulmonary ECs, such as oxidative injury and insulin resistance (Lane et al., 2011; Fessel et al., 2012; West et al., 2013). BMPR2 mutations also render pulmonary ECs susceptible to hypoxia- or inflammation-induced PH dysfunction (Song et al., 2005, 2008; Frank et al., 2008). Moreover, endothelin-1 upregulated by BMPR2 mutations contributes to PH pathogenesis (Star et al., 2013). Recent studies suggest that endothelial-to-mesenchymal transition plays critical roles in PH (Arciniegas et al., 2007; Ranchoux et al., 2015; Xiong 2015). The accumulation of knowledge in this area will spur new interest for both scientists and clinicians in the development of endothelial BMPR2 signaling-associated treatment. A comprehensive list of BMPR2 mutations has been summarized (Machado et al., 2006); however, their exact roles in diverse temporospatial contexts of complex PH remain unclear, such as gender bias (Liu et al., 2012). To address these issues scientists need the revelation of more secrets of endothelial BMPR2 signaling. It will be interesting to follow how the balancing act of endothelial BMPR2 signaling in treatment of PH is fine-tuned.

## Footnotes

The author is grateful for the support by the National Institutes of Health (NIH) Intramural Program and the Leducq Foundation. The author thanks NIH Fellows Editorial Board and NIH Library Writing Center for editorial assistance.

Jianhua Xiong declares that he has no conflict of interest. This article does not contain any studies with human or animal subjects performed by the author.
